# Supplementary material for: Nanodroplet processing platform for deep and quantitative proteome profiling of 10–100 mammalian cells
Source: Nat Commun. 2018 Feb 28;9:882. doi: 10.1038/s41467-018-03367-w (PMC5830451; doi:10.1038/s41467-018-03367-w)
Supplement: Supplementary file 1 — Supplementary information [file 41467_2018_3367_MOESM1_ESM.docx]

**Nanodroplet processing platform for deep and quantitative proteome profiling of 10-100 mammalian cells**

Zhu *et al.*

**Supplementary Figure 1**. Procedures for nanoPOTS chip fabrication and surface modification.

**Supplementary Figure 2**. Robotic platform for nanowell-based sample preparation.

**Supplementary Figure 3**. Peptide and protein identification from three blank control samples including SPE and LC columns, sample preparation reagents, and cell supernatant. To evaluate cross contamination from the SPE and LC columns, Buffer A was directly injected into SPE for LC-MS (blue). To evaluate cross contamination from sample preparation reagents, PBS buffer instead of cells was dispensed into nanowells followed by all proteomic processing steps (orange). To evaluate cross contamination from cell supernatant, a cell suspension with a concentration of ~200 cells/µL was centrifuged at 285 ×g for 10 min. The supernatant was dispensed into nanowells followed by all proteomic processing steps (gray). All these experiments were run after ~100-cell samples.

**Supplementary Figure 4**. (a) Images of 12, 42, and 139 HeLa cells in nanowells and (b) their corresponding base peak chromatograms.

**Supplementary Figure 5**. Evaluation of trypsin digestion efficiency. Percentages of full tryptic peptides and peptides with missed cleavage sites for samples with cell numbers from 10 to 141.

**Supplementary Figure 6**. (a–b) Overlap of protein groups identified from three cell loading groups with (a) MS/MS only method, and (b) combined MS/MS and MBR method. (c–d) Overlap of protein groups identified from similar cell loadings of 10, 12, and 14 cells with (c) MS/MS only method, and (d) combined MS/MS and MBR method.

**Supplementary Figure 7**. Evaluation of the performance of Match Between Runs in MaxQuant using an open source quality control software “PTXQC”.^6^ (a) Heatmap overview of 23 different quality metrics. The overall quality of MBR was shown in column “EVD: MBR – alignment” and “EVD: MBR - ID Transfer”. (b) The quality of retention time correction for all datasets. (c) The quality of ID-transfer for all datasets.

**Supplementary Figure 8**. Distribution of (a) mass error and (b) retention time difference of peptides identified from MS/MS to estimate the background level. (c) Distribution of mass error of peptides identified from Match Between Runs.

**Supplementary Figure 9**. Quantifiable numbers of (a) peptides, (b) protein groups with summed intensities and (c) LFQ intensities for three cell loading groups. Peptides and protein groups having intensities in all 3 samples with similar cell numbers were counted as quantifiable identifications.

**Supplementary Figure 10**. Pairwise correlation analysis of any two samples in peptide intensity level with cell loadings groups of (a) 10–14 cells, (b) 37–45 cells, and (c) 137–141 cells.

**Supplementary Figure 11**. Pairwise correlation analysis of any two samples in protein intensity level with cell loadings groups of (a) 10–14 cells, (b) 37–45 cells, and (c) 137–141 cells. LFQ intensity generated from MaxQuant was used for protein intensity calculation.


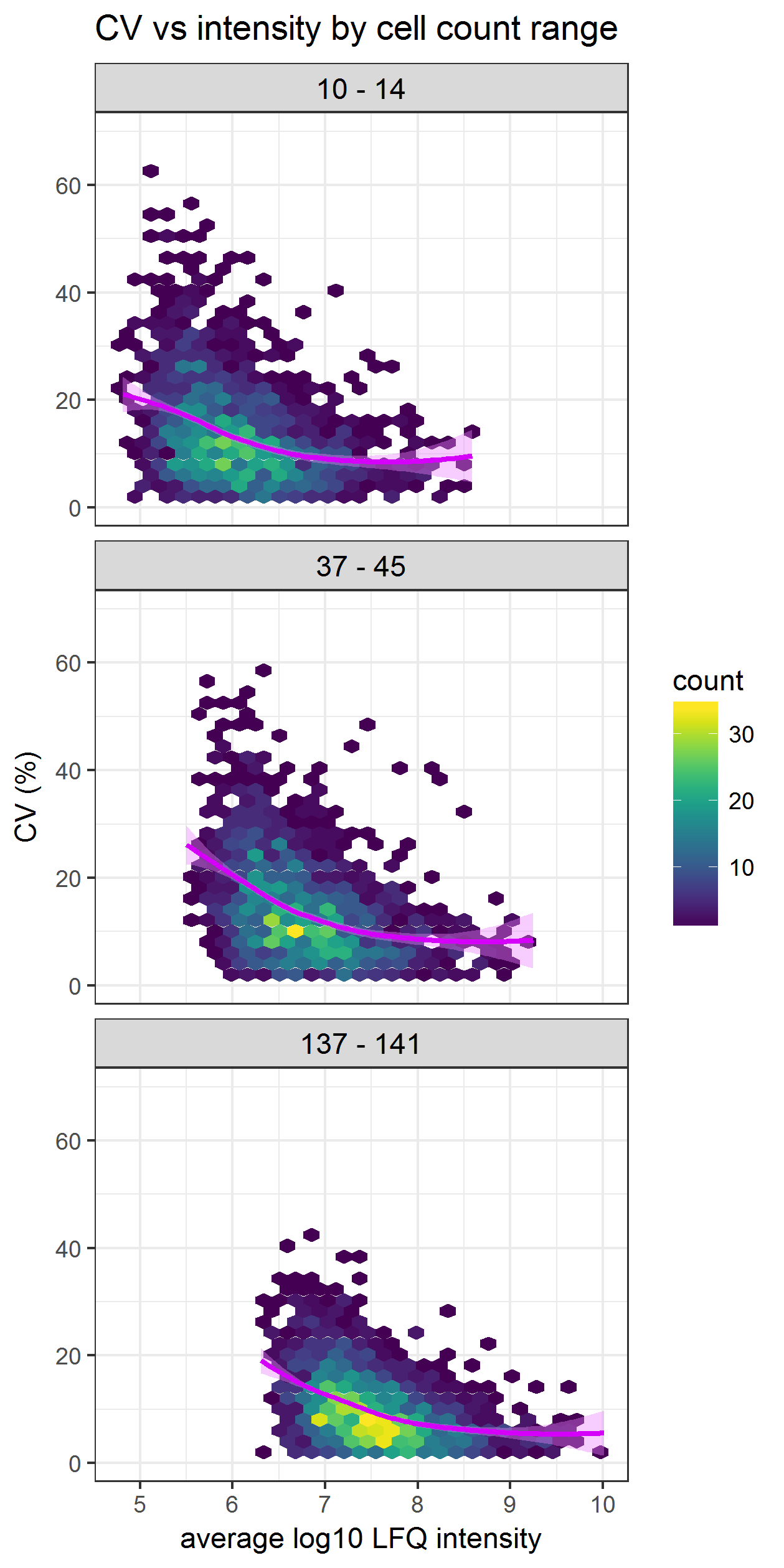


**Supplementary Figure 12**. The distributions of CVs versus average protein intensities for different cell loadings. Running average with standard deviations were indicated as pink lines.

**Supplementary Figure 13**. Performance on reproducibility with label free quantification. Boxplots showing the distributions of (a1, b1) coefficients of variation and (a2, b2) log intensities at (a1, a2) peptide and (b1, b2) protein level for three cell loading groups. Center lines show the medians; small squares show the means; box limits indicate the 25th and 75th percentiles; whiskers extend 1.5 times the interquartile range from the 25th and 75th percentiles. LFQ intensities generated by MaxQuant were used for protein quantification.

**Supplementary Figure 14**. Images of 9 islet sections from a non-diabetic donor (C_1 to C_9). Scale bar, 500 µm.

**Supplementary Figure 15**. Comparison of Gene Ontology annotations for Cellular Component showing the protein identified from nanoPOTS and SNaPP platforms.^9^ In nanoPOTS platform, datasets were generated 9 slices of LCM islets. In the SNaPP platform, datasets were generated from triplicate runs of over 100 islets.

**Supplementary Figure 16**. (a) Volcano Plot of differentially expressed proteins in comparative analyses between control and T1D islets. 304 proteins (blue points) were found to have significant differences with a fold change > 2 and a q-value (FDR) cutoff of 0.02 based on the Benjamini-Hochberg procedure. Note that we also applied the same statistical procedure on the same dataset without imputation of missing data points. 321 proteins were found to have significant differences with a FDR cutoff of 0.02 (without considering fold-changes), and 111 proteins were common between the two analyses. Among the common significant proteins, 56 have at least one missing data across the 18 single islet samples. (b) Comparison of the fold change of the 56 proteins with missing data points with and without imputation. The good correlation of fold changes between analyses with and without imputation suggests the imputation approach is largely valid. Significant proteins identified using these analyses are provided as Supplementary Data 1.

**Supplementary Table 1**. Previously published protein identification results from studies of 2000 or fewer mammalian cells^*^

| Cell number | Cell type | Identified protein number | Sample preparation method |
| --- | --- | --- | --- |
| 100 | DLD-1 | 635 | High temperature trypsin digestion^1^ |
| 250 | DLD-1 | 759 | High temperature trypsin digestion^1^ |
| 500 | DLD-1 | 1060 | High temperature trypsin digestion^1^ |
|  | MCF-7 | 187 | Acetone precipitation^2^ |
|  | HeLa | 905 | FASP^3^ |
| 1000 | MCF-7 | 271 | Acetone precipitation^2^ |
|  | HeLa | 1536 | FASP^3^ |
|  | MCF-7 | 2512 | AFA assisted cell lysis^4^ |
| 2000 | HEK 239T | 1270 | Spin tip^5^ |
|  | MCF-7 | 3370 | AFA assisted cell lysis^4^ |

^*^Reports in which samples were prepared in bulk and subsequently analyzed with a small number of cell equivalents are not included in this table.

**Supplementary Table 2**. Copy numbers per HeLa cell for proteins identified from 10-14 cells^*^

| Protein Name | Gene Names | Protein copy number per HeLa cell |
| --- | --- | --- |
| Pre-mRNA-splicing regulator WTAP | WTAP | 49,143 |
| ATPase family AAA domain-containing protein 2 | ATAD2 | 63,835 |
| Poly [ADP-ribose] polymerase 4 | PARP4 | 63,971 |
| Carbonyl reductase [NADPH] 3 | CBR3 | 79,823 |
| Endoplasmic reticulum lipid raft-associated protein 2 | ERLIN2 | 149,867 |
| THO complex subunit 1 | THOC1 | 204,962 |
| 28S ribosomal protein S23, mitochondrial | MRPS23 | 223,198 |
| Hepatocellular carcinoma-associated antigen 59 | C9orf78 | 265,003 |
| COP9 signalosome complex subunit 5 | COPS5 | 323,791 |
| Nucleoprotein TPR | TPR | 357,637 |
| AFG3-like protein 2 | AFG3L2 | 369,737 |
| 28S ribosomal protein S35, mitochondrial | MRPS28 | 422,825 |
| Prefoldin subunit 1 | PFDN1 | 476,849 |
| Cytosolic acyl coenzyme A thioester hydrolase | ACOT7 | 512,746 |
| Cytochrome b-c1 complex subunit 1, mitochondrial | UQCRC1 | 1,022,450 |
| 26S protease regulatory subunit 6A | PSMC3 | 1,062,048 |
| Eukaryotic translation initiation factor 3 subunit 6 | EIF3E | 1,067,627 |
| FACT complex subunit SSRP1 | SSRP1 | 1,095,695 |
| Ras GTPase-activating-like protein IQGAP1 | IQGAP1 | 1,296,511 |
| SRA stem-loop-interacting RNA-binding protein, | SLIRP | 1,397,500 |
| Purine nucleoside phosphorylase | PNP | 1,555,814 |
| Heat shock 70 kDa protein 4 | HSPA4 | 1,646,549 |
| 14-3-3 protein sigma | SFN | 1,870,568 |
| Flap endonuclease 1 | FEN1 | 2,019,699 |
| Enoyl-CoA hydratase, mitochondrial | ECHS1 | 2,105,336 |
| Transitional endoplasmic reticulum ATPase | VCP | 2,719,254 |
| Fatty acid synthase | FASN | 3,536,145 |
| T-complex protein 1 subunit beta | CCT2 | 4,479,130 |
| ATP synthase subunit beta, mitochondrial | ATP5B | 4,511,967 |
| Peroxiredoxin 6 | PRDX6 | 8,781,079 |
| Peptidyl-prolyl cis-trans isomerase B | PPIB | 10,502,199 |
| Vimentin | VIM | 22,886,339 |

^*^The copy numbers were obtained from the PrEST-SILAC method.^7^

**Supplementary Table 3**. Calculation of cell number and islet equivalents with islet areas^*^

|  | Islet Area (µm^2^) | Islet volume (µm^3^) | Cell Number | Islet equivalents (IEQ) |
| --- | --- | --- | --- | --- |
| C_1 | 30197 | 301973 | 266 | 0.17 |
| C_2 | 16200 | 162004 | 143 | 0.09 |
| C_3 | 21286 | 212862 | 188 | 0.12 |
| C_4 | 11133 | 111330 | 98 | 0.06 |
| C_5 | 14235 | 142354 | 125 | 0.08 |
| C_6 | 22428 | 224280 | 198 | 0.13 |
| C_7 | 10365 | 103654 | 91 | 0.06 |
| C_8 | 15186 | 151860 | 134 | 0.09 |
| C_9 | 21474 | 214738 | 189 | 0.12 |
| T1D_1 | 28585 | 285846 | 168 | 0.16 |
| T1D_2 | 51576 | 515761 | 303 | 0.29 |
| T1D_3 | 68343 | 683431 | 402 | 0.39 |
| T1D_4 | 47668 | 476680 | 280 | 0.27 |
| T1D_5 | 29607 | 296072 | 174 | 0.17 |
| T1D_6 | 25004 | 250035 | 147 | 0.14 |
| T1D_7 | 28113 | 281127 | 165 | 0.16 |
| T1D_8 | 24445 | 244446 | 144 | 0.14 |
| T1D_9 | 77159 | 771593 | 454 | 0.44 |

^*^Reference 8; Islets from C_1 to C_9 were from a non-diabetic donor; Islets from T1D_1 to T1D_9 were from a type 1 diabetic donor.

# Supplementary References

1. Chen, Q., Yan, G., Gao, M. & Zhang, X. Ultrasensitive Proteome Profiling for 100 Living Cells by Direct Cell Injection, Online Digestion and Nano-LC-MS/MS Analysis. *Anal. Chem.* **87,** 6674–6680 (2015).

2. Wang, N., Xu, M., Wang, P. & Li, L. Development of mass spectrometry-based shotgun method for proteome analysis of 500 to 5000 cancer cells. *Anal. Chem.* **82,** 2262–2271 (2010).

3. Wiśniewski, J. R., Ostasiewicz, P. & Mann, M. High recovery FASP applied to the proteomic analysis of microdissected formalin fixed paraffin embedded cancer tissues retrieves known colon cancer markers. *J. Proteome Res.* **10,** 3040–3049 (2011).

4. Li, S. *et al.* An Integrated Platform for Isolation, Processing, and Mass Spectrometry-based Proteomic Profiling of Rare Cells in Whole Blood. *Mol. Cell. Proteomics* **14,** 1672–1683 (2015).

5. Chen, W. *et al.* Simple and Integrated Spintip-Based Technology Applied for Deep Proteome Profiling. *Anal. Chem.* **88,** 4864–4871 (2016).

6. Bielow, C., Mastrobuoni, G. & Kempa, S. Proteomics Quality Control: Quality Control Software for MaxQuant Results. *J. Proteome Res.* **15,** 777–787 (2016).

7. Zeiler, M., Straube, W. L., Lundberg, E., Uhlen, M. & Mann, M. A Protein Epitope Signature Tag (PrEST) Library Allows SILAC-based Absolute Quantification and Multiplexed Determination of Protein Copy Numbers in Cell Lines. *Mol. Cell. Proteomics* **11,** O111.009613 (2012).

8. Pisania, A. *et al.* Quantitative analysis of cell composition and purity of human pancreatic islet preparations. *Lab. Invest.* **90,** 1661–1675 (2010).

9. Huang, E. L. *et al.* Snapp: Simplified nanoproteomics platform for reproducible global proteomic analysis of nanogram protein quantities. *Endocrinology* **157,** 1307–1314 (2016).
