# Supplementary material for: Nanodroplet processing platform for deep and quantitative proteome profiling of 10–100 mammalian cells
Source: Nat Commun. 2018 Feb 28;9:882. doi: 10.1038/s41467-018-03367-w (PMC5830451; doi:10.1038/s41467-018-03367-w)
Supplement: Supplementary file 3 — Descriptions of Additional Supplementary Files [file 41467_2018_3367_MOESM3_ESM.pdf]

Description of Additional Supplementary Files:

File Name: Supplementary Data 1
Description: Supplementary Data 1 contains three excel spreadsheets: 1) List of significant proteins between T1D and control islets identified with imputation of missing data; 2) List of significant proteins identified without imputation of missing data; 3) Significant proteins common to both analyses.
